# Die elektronische Patientenakte im Krankenhausinformationssystem

**DOI:** 10.1007/s00347-020-01048-y

**Published:** 2020-03-18

**Authors:** Armin Mir Mohi Sefat, Katrin Patermann, Lars von Ohlen, Andrea Kühr, Mahdy Ranjbar, Werner Pauls, Rudolf Dück, Salvatore Grisanti

**Affiliations:** 1 Klinik für Augenheilkunde, Universitätsklinikum Schleswig-Holstein - Campus Lübeck, Ratzeburger Allee 160, 23538 Lübeck, Deutschland; 2 UKSH Gesellschaft für IT Services mbH, Maria-Goeppert-Straße 7, Lübeck, 23562 Lübeck Deutschland; 3 UKSH Stabsstelle IT, Maria-Goeppert-Str. 7, Lübeck, 23562 Deutschland

**Keywords:** Digitalisierung, Krankenakte, Bildgebung, Dokumentation, Informationstechnologie, Elektronische Patientenakte, Krankenakte, Digitization, Patient records, Imaging, Documentation, Information technology

## Abstract

**Hintergrund:**

Die Einführung einer elektronischen Patientenakte (EPA) im Bereich der Ophthalmologie stellt für viele Kliniken eine Herausforderung dar. Obwohl alle Kliniken ein Krankenhausinformationssystem (KIS) besitzen, sind die wenigsten dieser Systeme für die komplexe ophthalmologische Patientenakte gerüstet.

**Methodik:**

Wir berichten über die Implementierung einer ophthalmologischen EPA innerhalb des vorherrschenden KIS (Agfa-ORBIS; Agfa HealthCare GmbH, Bonn). Dabei werden sowohl die digitale Aktenführung als auch die Anbindung der vorhandenen Untersuchungsgeräte dargestellt.

**Ergebnisse:**

Die von uns entwickelte EPA wird im klinischen Alltag seit 2009 genutzt und seitdem kontinuierlich weiterentwickelt. Durch eine enge Zusammenarbeit mit der IT-Abteilung konnten alle Untersuchungsgeräte digital angebunden werden und ein papierloses Arbeiten ermöglichen und die Nachteile der Papierakte vermeiden.

**Diskussion:**

Die Nutzungsmöglichkeiten einer in das vorhandene KIS implementierten EPA sind vielfältig. Durch solch ein System kann eine lückenlose, fächerübergreifende und ubiquitäre Dokumentation erfolgen. Die Alternative stellt die Anschaffung eines Drittsystems dar, welches sowohl durch eine Schnittstelle mit dem Hauptsystem verbunden werden muss als auch deutliche höhere Anschaffungs- und Erhaltungskosten aufweist.

## Abstract (en)

**Background:**

The implementation of an electronic patient health record (EHR) system in the field of ophthalmology can be a challenging project for many hospitals. Although all hospitals have a hospital information systems (HIS), these systems are often not capable of managing the complexity of ophthalmological patient records.

**Methods:**

This article reports the implementation of an ophthalmological EHR into the pre-existing HIS (Agfa-ORBIS; Agfa HealthCare GmbH, Bonn, Germany). The digital record management and the connections to the peripheral examination devices are presented.

**Results:**

The EHR developed by us has been in clinical use since 2009 and is subject to continuous further development. Through close cooperation with the IT department, all examination devices could be digitally connected, enabling paperless work and avoiding the drawbacks of paper files.

**Discussion:**

The advantages of an EHR implemented into the existing HIS are manifold. Through such a system a complete, interdisciplinary and ubiquitous documentation can take place. The alternative is the acquisition of a third system, which must be connected by an interface with the main system and has significantly higher acquisition and maintenance costs.

Die Einführung und Nutzung einer elektronischen Patientenakte ist ein wichtiger Meilenstein im Zuge der Digitalisierung des Gesundheitssystems. Die Umstellung von der Papierakte ist im Praxisalltag längst vollzogen, stellt aber für viele Kliniken noch eine große Herausforderung dar. Häufig ist das vorherrschende Krankenhausinformationssystem (KIS) nicht für die komplexe ophthalmologische Akte angepasst, sodass kostenintensive Drittsysteme angeschafft werden. In diesem Beitrag soll gezeigt werden, wie in enger Zusammenarbeit mit der hauseigenen IT-Abteilung eine komplexe elektronische Patientenakte für die Ophthalmologie direkt im bestehenden KIS umgesetzt wurde.

## Hintergrund

Die Digitalisierung im Krankenhaus bedingt die Einrichtung einer elektronischen Patientenakte (EPA). Deutschlandweit sind alle Krankenhäuser mit Krankenhausinformationssystemen (KIS), die primär verwaltungstechnische Aufgaben erfüllen, ausgestattet. Nicht selten erfolgt die Erfassung medizinischer Daten aber weiterhin in Form einer Papierakte. Die damit verbundenen Nachteile liegen im wahrsten Sinne des Wortes auf der Hand. Die Papierakte ist häufig unübersichtlich, schwer lesbar, bindet räumliche und personelle Ressourcen und ist in der vollvernetzten Welt des 21. Jahrhunderts nicht mehr zeitgemäß. Die EPA ist wichtig und notwendig zur Verbesserung der Effizienz, der Sicherheit und der Qualität der Patientenversorgung [5, 10].

In der Augenheilkunde ist der Bedarf einer geordneten, übersichtlichen und vollständigen Darstellung der unterschiedlichsten Untersuchungen und Befunde besonders groß [4]. Die hohe Zahl an Patientenkontakten sowie die Häufung chronischer und komplexer Krankheitsverläufe machen eine schnelle Orientierung in der Patientenakte unverzichtbar. Zudem ist die Augenheilkunde im zunehmenden Maße von neuen Imaging-Methoden geprägt und ist insgesamt stark von einer guten Visualisierung abhängig. In den vergangenen 2 Jahrzehnten wurden deshalb von unterschiedlichen Anbietern EPA für die Ophthalmologie (z. B. IFA Systems AG, Frechen; Arztservice Wente GmbH, Darmstadt; Medisoft Ltd., Leeds, UK) entwickelt und kommerziell angeboten. Diese Systeme wurden primär für die Praxis und für die Versorgung ambulanter Patienten entwickelt.

Einige Kliniken nutzen diese Systeme im Sinne einer Hybridlösung mit dem bestehenden KIS. Hierbei handelt es sich aber um einen Kompromiss mit Hindernissen. Anders als in der ambulanten Praxis sind für ein Haus der Maximalversorgung die interdisziplinäre Arbeit und die uneingeschränkte Verfügbarkeit der erhobenen Befunde unerlässlich. Zudem müssen rechtliche Aspekte und die Datensicherheit und Datensicherung kritisch geprüft werden und gewährleistet sein [1].

Das Arbeiten mit einer einzigen Plattform vereinfacht die Vernetzung, die digitale Darstellung in tiefere Organisationsstrukturen und die interdisziplinäre Zusammenarbeit. Aus diesen Gründen entschieden wir uns für die EPA im KIS und begannen 2009 an der Augenklinik des Universitätsklinikums Schleswig-Holstein, Campus Lübeck (UKSH, Lübeck) eine EPA für die Ophthalmologie in das bestehende KIS (ORBIS, Agfa HealthCare GmbH, Bonn) einzurichten. Diese sollte die Vorteile bestehender kommerzieller Lösungen, aber nicht die damit verbundenen Nachteile haben. In diesem Artikel sollen das System vorgestellt sowie die Vorteile gegenüber der Insellösung eines Drittsystems hervorgehoben werden.

## Methodik

### Ausgangslage

Im Jahr 2008 wurde das klinische Informationssystem ORBIS (Agfa HealthCare GmbH, Bonn) vom UKSH für beide Standorte (Kiel und Lübeck) angeschafft. Zum Zeitpunkt der Anschaffung wurden die grundlegenden Funktionen des Systems wie die Patientenverwaltung und das Erlösmanagement genutzt. Die einzelnen Fachdisziplinen inklusive der Ophthalmologie arbeiteten weiterhin mit einer Papierakte.

Aufgrund der klaren Vorteile einer EPA im KIS wurde im Jahr 2009 sukzessiv begonnen, die Papierakte an der Universitätsaugenklinik in Lübeck zu ersetzen und eine EPA in das bestehende KIS zu implementieren.

Die am UKSH vorhandene Version des KIS (ORBIS-NICE) bietet die Möglichkeit, Untersuchungsformulare in digitaler Form anzulegen. Das System kann sowohl die ambulanten als auch die stationären Patienten verwalten, die OP-Planung und Terminvergabe steuern und integriert fächerübergreifende Befunde.

### Technische Ausstattung und Geräteanbindung

Alle Arbeitsplätze wurden mit einem PC (Windows, Microsoft, Redmont, WA, USA) und einem Scanner ausgestattet, welche direkt an das zentrale KIS (ORBIS) angeschlossen sind. Das zentrale KIS verwaltet die Daten von ca. 400.000 Fällen, die jährlich im UKSH vorstellig werden. Die zentrale Speicherarchitektur ist als Cluster mit 6 einzelnen Isilonsystem realisiert, die sich zu jeder Zeit abgleichen. Ein Backup dieses Bereiches kann zusätzlich auf ein Backupsystem Datadomain gesichert werden.

Die in der Ophthalmologie verwendeten diagnostischen Geräte (z. B. optische Kohärenztomographie, Angiographie, Sonographie, Elektrophysiologie) wurden mit dem zentralen Server verbunden und die Datenbanken übertragen.

**Figure 1:**
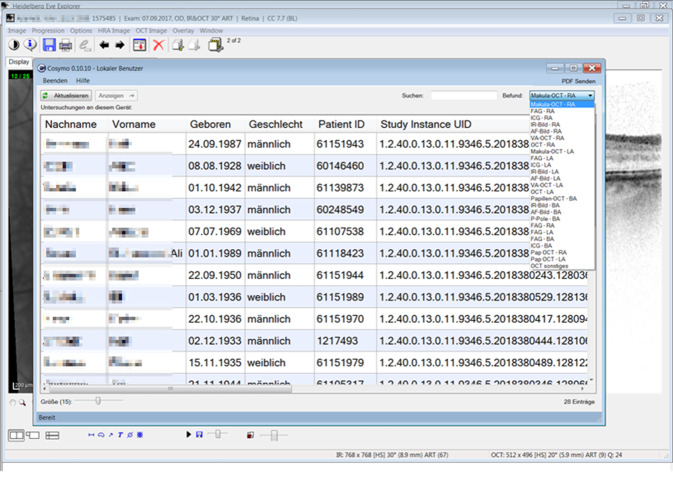

Um diese Anbindung zu realisieren, wurde gemeinsam mit der UKSH Gesellschaft für IT-Services mbH (UKSH-ITSG) ein Tool entwickelt (UKSH-ITSG, Schnittstellen-Tool) welches auf nahezu allen Drittgeräten installiert werden kann. Das Tool empfängt die Patienteninformationen über eine DICOM (Digital Imaging and Communications in Medicine)-Worklist, welche anschließend die Informationen über verschiedene HL7 (Health Level 7) MDM(Medical Document Management)/ORU(Observation Result Unsolicited)-Schnittstellen mit ORBIS kommuniziert (Abb. 1). So können die an den Untersuchungsgeräten erhobenen Befunde als PDF oder JPEG direkt zu dem jeweiligen Patienten in das ORBIS eingepflegt und anschließend an jedem Arbeitsplatz eingesehen werden.

Alle ophthalmologischen Daten zu einem Patienten sind digital gespeichert, chronologisch archiviert und mit der Leistungserfassung verknüpft, schnell aufrufbar, interdisziplinär sichtbar und nicht lokal begrenzt, sodass diese auch im Rahmen von Konsilen außerhalb der „Mutterklinik“ einsehbar und ergänzbar sind.

Bei Untersuchungsgeräten mit einer komplexen Benutzersoftware (z. B. Heidelberg Eye Explorer (Heidelberg Engineering GmbH, Heidelberg), Haag-Streit Eye-Suite (Haag-Streit Holding AG, Könitz, Schweiz), Oculus Pentacam Software (Oculus Optikgeräte GmbH, Wetzlar), deren Funktionalität weit über die Darstellung eines einzelnen Ausdruckes hinausgeht, ist es möglich, über eine Integration in das KIS zu dem jeweiligen Patienten diese Software zu öffnen (z. B. Heidelberg Eye Explorer) und die Funktionalität des originären Systems in vollem Umfang zu nutzen. Dies geschieht über einen Webaufruf mit Zugriff auf die zentral verwaltete Software. Die von diesen Systemen genutzten Datenbanken wurden auf gesicherten und gespiegelten Servern angelegt.

### Rechtliche Aspekte und Datensicherheit

Die von uns erhobenen Daten unterliegen der Datenschutzgrundverordnung (EU-DSGVO; Verordnung 2016/679). Durch eine Einwilligung bei Aufnahme in das System geben die Patienten dem UKSH die Genehmigung zur Datenerfassung. Alle erhobenen Daten unterliegen dem Berechtigungskonzept des KIS, sodass im Gegensatz zur Hybridlösung kein zusätzliches Datenschutzkonzept erarbeitet werden muss. Im ORBIS wird jeder Aufruf eines Dokumentes zeitlich dokumentiert und archiviert. Zudem werden die Daten in der zentralen Serverfarm gesichert und archiviert.

Ein weiterer wichtiger rechtlicher Aspekt ist die Einverständniserklärung zu operativen Eingriffen. ORBIS hat hier eine Schnittstelle zu E‑ConsentPro (Thieme Compliance GmbH, Erlangen) und erstellt personalisierte Patientenaufklärungen. Aktuell werden diese noch von den Patienten physisch signiert und anschließend eingescannt. Ein Pilotprojekt zur Signierung mittels eines digitalen Signierpads wurde im UKSH inzwischen erfolgreich durchgeführt und das System auf weitere Kliniken übertragen.

## Ergebnisse

Beginnend im Jahr 2009 wurde eine spezialisierte ophthalmologische Patientenakte im ORBIS angelegt. Das Modul wurde durch die Formularentwicklung der UKSH ITSG in enger Zusammenarbeit mit der Augenklinik (ohne Beteiligung der Fa. Agfa) entwickelt. Die UKSH ITSG leistet auch den IT-Support. Die verwendeten Module von ORBIS sind BDOK, LSTM und die Synopsis. Im Folgenden sollen der Aufbau der Patientenakte und deren Funktionalität dargestellt werden.

### Aufbau der Patientenakte

**Figure 2:**
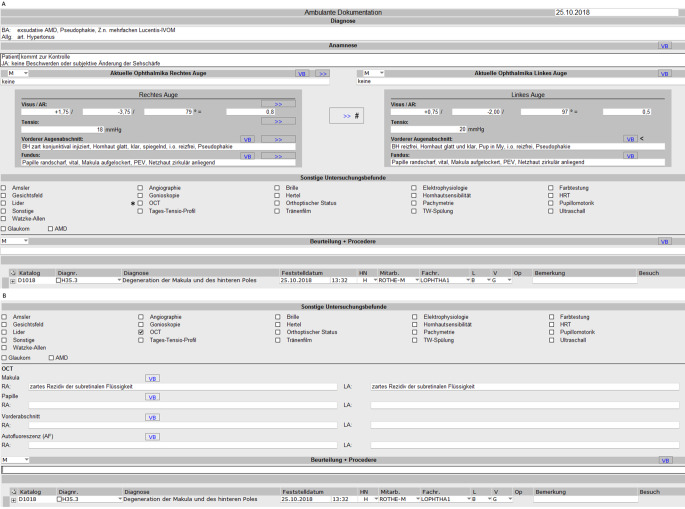

**Figure 3:**
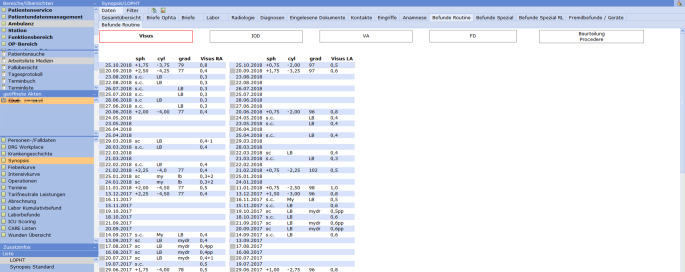

Die EPA besteht im Wesentlichen aus 2 Einheiten: dem Befundformular (Abb. 2) und der Synopsis (Abb. 3).

Das Behandlungsformular folgt der üblichen medizinischen Informationserfassung und beinhaltet die Diagnosen, die Anamnese und die Medikation. Die Erfassung ophthalmologischer Befunde folgt der Systematik, die sich in der Augenheilkunde über Jahrzehnte etabliert hat. Die klinische Befundung ist in einen Sektor für den Vorder- und einen für den Hinterabschnitt unterteilt. Vorbefunde können eingesehen oder übernommen werden. Die Befunderhebung erfolgt separat für das rechte und linke Auge und wird auch in allen anderen Bereichen der EPA so dargestellt. Um eine visuelle Überfrachtung zu vermeiden, öffnet sich die Befundbox für spezielle Untersuchungen nur, wenn die entsprechende Checkbox (Abb. 2a Sternchen) aktiviert wird (Abb. 2b). Sämtliche erhobenen Befunde lassen sich befundspezifisch in der Synopsis aufrufen (Abb. 3).

Abschließend kann unter „Beurteilung/Prozedere“ der Befund und das weitere Prozedere zusammengefasst werden.

Die Behandlungsformulare werden mitarbeiterspezifisch dokumentiert und digital signiert. Der Mitarbeiter loggt sich über sein Passwort oder über einen Kartenscanner ein. Letzteres ermöglicht, bei Computerwechsel den an anderer Stelle geöffneten Fall ohne Zeitverlust durch erneutes Aufrufen fortzusetzen. Zudem arbeitet das ORBIS mit Zeitstempeln, so kann über die „Dokumentenhistorie“ jederzeit eingesehen werden, welcher Mitarbeiter zu welchem Zeitpunkt das Formular geöffnet oder bearbeitet hat (dies gilt für alle Dateien im ORBIS). So kann eine lückenlose, mitarbeiterspezifische und rechtssichere Dokumentation gewährleistet werden.

**Figure 4:**
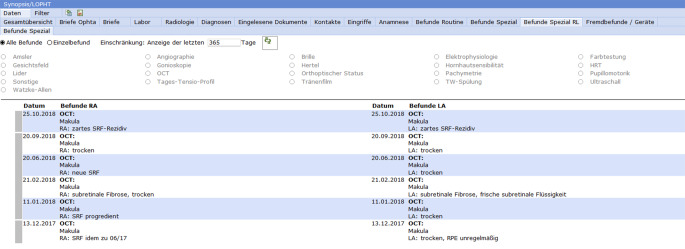

Die Synopsis (Abb. 3) dient dem chronologischen Überblick. Sie wurde für unsere Klinik als spezialisierte ophthalmologische Synopsis modifiziert. Über sie kann die gesamte Patientendokumentation eingesehen werden. Dort werden aktuelle Daten, Voruntersuchungen, Altbefunde und von extern eingescannte Formulare dargestellt. Hierfür gibt es unterschiedliche Untereinheiten der Synopsis. Zentrales Element sind die „Befunde Routine“. Hier können der Visus, der Intraokulardruck (IOD), der Augenvorderabschnitt sowie der Fundus und die Beurteilung bzw. das Prozedere über den zeitlichen Verlauf übersichtlich eingesehen werden (Abb. 4). Von dieser Ansicht kann man jedes zugrunde liegende Befundformular direkt aufrufen. Die chronologische Auflistung erlaubt deshalb bei langen komplizierten Verläufen nicht nur einen schnellen Befundüberblick, sondern auch einen Überblick über die Entscheidungsfindung bzw. Strategieänderung und deren Gründe.

Folgende weitere Untereinheiten mit den zugeordneten Daten sind in der Synopsis der ophthalmologischen EPA angelegt und einzeln abrufbar:

- Gesamtübersicht: alle zu dem Patienten vorhandenen Dokumente. Mittels Filter und Suchfunktion können gezielt Dokumente aufgerufen werden;
- Briefe: alle am UKSH erstellten Briefe;
- Labor: alle im Klinikum generierten Labordaten;
- Radiologie: Zugriff auf die radiologischen Befunde und das IMPAX;
- Diagnosen;
- eingelesene Dokumente: alle gescannten Unterlagen. Mittels Filter und Suchfunktion können gezielt Dokumente aufgerufen werden;
- Kontakte;
- Eingriffe: alle durchgeführten Eingriffe mit direkter Möglichkeit, den OP-Bericht aufzurufen;
- Anamnese: Erstanamnese und aktuelle Anamnese (Stammblatt);
- Befunde Routine: chronologische Darstellung von Visus, IOD, VA, FD und Beurteilung/Prozedere;
- Befunde Spezial: chronologische Übersicht der Befunde weiterer Untersuchungsgeräte (z. B. OCT, Angiographie, Gesichtsfeld etc.). Mittels Filter gezielte Darstellung der Befunde einzelner Geräte möglich;
- Fremdbefunde/Geräte: Ansicht der über das Schnittstellen-Tool eingepflegten Befunde. Mittels Filter und Suchfunktion können gezielt Dokumente aufgerufen werden.

**Figure 5:**
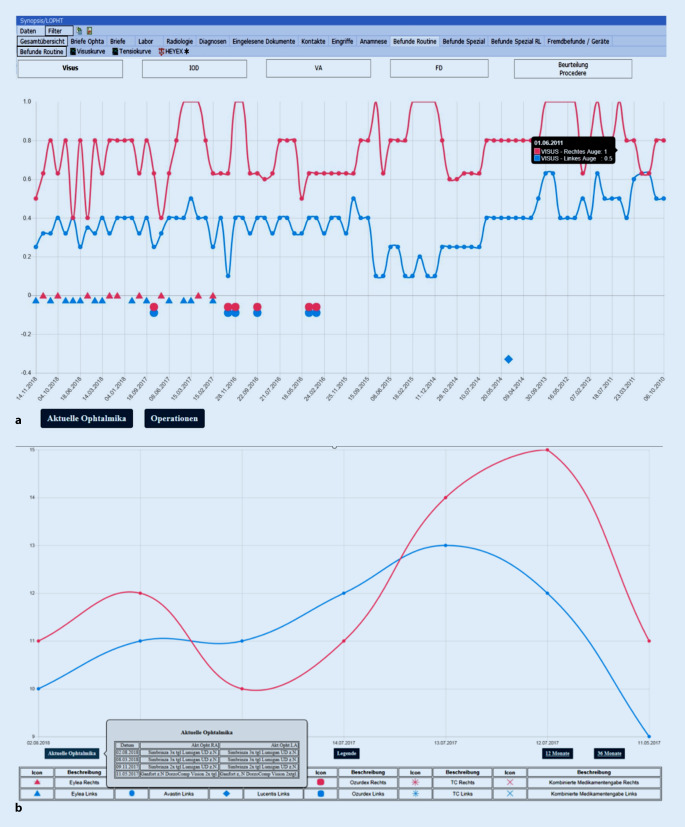

Die Synopsis ermöglicht per Fremdaufruf den direkten Zugriff und volle Funktionalität auf die Benutzersoftware von ophthalmologischer Spezialsofware wie z. B. den Heidelberg Eye Explorer (Heidelberg Engineering GmbH, Heidelberg). Diese apparativen Untersuchungsergebnisse und Analysetools der Gerätesoftware sind für die Festlegung der therapeutischen Vorgehensweise immanent wichtig. Weiter ist es möglich, Visus- oder IOD-Kurven mit den chronologisch erfassten Eingriffen (z. B. IVOM oder OP) oder mit den applizierten Ophthalmika darzustellen (Abb. 5).

### Terminbuch und Tageslisten

Eine der Basisfunktionen des ORBIS ist die Möglichkeit der Terminbuchführung. Jede Unterabteilung unserer Klinik hat ihr eigenes Terminbuch und kann für den jeweiligen Tag eine Tagesliste mit den erwarteten Patienten erstellen. Wenn ein Patient die Klinik betritt, wird er in das System aufgenommen, und es kann ab diesem Zeitpunkt auch nachvollzogen werden, wann er da war, wann er von welchem Mitarbeiter behandelt worden ist und wann die Untersuchung abgeschlossen wurde. Zudem kann man zu dem jeweiligen Patienten auch dessen individuellen Terminkalender öffnen. Die individuelle Patientenbewegung über die einzelnen Stationen (z. B. Vorderabschnittuntersuchung oder Fluoreszenzangiographie) ist anhand der durchgeführten Leistung erkennbar. Der zeitliche Ablauf und die Aufenthaltsdauer in der Klinik sind anhand der initial erfassten Zeit erkennbar. Diese ist mit einem Ampelsystem belegbar, um unerwünschte Zeitüberschreitungen besser erkennbar zu machen.

### Fremdbefunde, Einverständniserklärungen und Skizzen

Alle vom Patienten mitgebrachten Unterlagen (z. B. Briefe, Überweisung) werden zentral an der Anmeldung als „Eingelesene Dokumente“ eingescannt und spezifisch benannt und stehen anschließend sofort in der Synopsis zur Verfügung. Für die Einverständniserklärungen zu geplanten Eingriffen verwenden wir das Programm E‑ConsentPro. Die digital erstellten Einverständniserklärungen müssen einmal ausgedruckt und anschließend nach der Aufklärung des Patienten und der ärztlichen Dokumentation der Aufklärung unterschrieben werden. Dieses Dokument wird eingescannt und dient der Dokumentation. Das Original wird den Patienten mitgegeben. Die Signierung mittels digitalem Signierpad wird derzeit eingeführt.

Über die Funktion „Eingelesene Dokumente“ besteht an jedem klinischen Arbeitsplatz auch die Möglichkeit, eigene Unterlagen (z. B. Fundusskizzen, OP-Checklisten) direkt zum jeweiligen Patienten einzuscannen.

### Stationsarbeit

ORBIS bietet die Möglichkeit der Darstellung einer Stationsgrafik. Diese enthält ein Signalsystem, um z. B. über eingegangene pathologische Laborergebnisse aufmerksam zu machen. Über diese Grafik lässt sich per „Rechtsklick“ die gesamte Funktionalität der EPA zu einem Patienten erreichen. Die Synopsis verschafft einen schnellen chronologischen Überblick über die Patientenakte. Man kann zu jeder Visite ein neues Behandlungsformular anlegen und dort über Mouseover den Befund des Vortages anzeigen lassen. Über die Synopsis kann auch die digitale Kommunikation mit der ORBIS-Fieberkurve stattfinden. Diese stellt im Prinzip den zentralen Arbeitsbereich der Patientendokumentation der Stationspflege dar und hat die papiergeführte Pflegekurve ersetzt. Sowohl die Pflegekräfte als auch das ärztliche Personal haben einen kompletten Einblick in die Synopsis und in die Fieberkurve. Die Schreibrechte sind entsprechend der jeweiligen Funktion des Mitarbeiters verliehen.

Über einen Button „Anordnungen“ können pflegespezifische Anordnungen durchgeführt werden (z. B. Lagerung, Blutentnahmen, Verbandswechsel). Diese Anordnungen tauchen bei der Pflege in der „Fieberkurve“ auf, können abgearbeitet werden und müssen ebenfalls mitarbeiterspezifisch dokumentiert werden. Die Medikamentenverordnungen werden am UKSH aktuell über MEONA (MEONA GmbH, Freiburg) durchgeführt und müssen ebenfalls sowohl von den Ärzten als auch von der Pflege mitarbeiterspezifisch dokumentiert werden (Anordnung/Verabreichung). ORBIS und MEONA stehen in direkter Kommunikation. So können z. B. Medikamentenpläne aus dem MEONA in das ORBIS eingefügt werden.

### OP-Bereich

Eine weitere Basisfunktion der EPA im KIS ist der OP-Plan. Patienten können über ein Anforderungsformular direkt auf den OP-Plan gesetzt werden. Hier bietet ORBIS auch die Möglichkeit, die jeweiligen Operationen mit den dazugehörigen ICD- und OPS-Codes vorzubelegen. Zusätzlich kann das für den Eingriff notwendige Instrumentarium und Verbrauchsmaterial hinterlegt werden. Hierdurch wird eine schnelle Dokumentation sowohl vonseiten der Pflege als auch von ärztlicher Seite ermöglicht.

Das OP-Personal überprüft am Anfang des OP-Tages alle elektronischen Patientenakten der geplanten Patienten auf Vollständigkeit von OP-relevanten Dokumenten (z. B. Einverständniserklärung oder Berechnung der einzusetzenden Intraokularlinse), um diese bei Fehlen rechtzeitig vor Einschleusung des Patienten einzufordern. Im OP-Plan lässt sich analog zur Stationsgrafik per „Rechtsklick“ die gesamte Funktionalität der EPA zu einem Patienten erreichen.

Die geplante OP-Dauer, der OP-Zeitpunkt und das OP-Team sind im Voraus festgelegt. Die OP-Koordination hat zudem den kompletten Überblick über die virtuellen Säle des gesamten Klinikums und kann anhand der Information zur Auslastung und zur Über- oder Unterschreitung der geplanten OP-Dauer aktiv die Saalzuweisung steuern und Engpässe abwenden. Zusätzlich kann aber auch jeder andere Mitarbeiter von seinem Arbeitsplatz außerhalb des OPs den aktuellen Status einer OP (Einleitung/chirurgische Maßnahmen/Ausleitung sind farblich codiert) oder ob es z. B. kurzfristige Änderungen des OP-Planes gegeben hat, einsehen und rechtzeitig Gegenmaßnahmen einleiten.

### Brieferstellung

**Figure 6:**
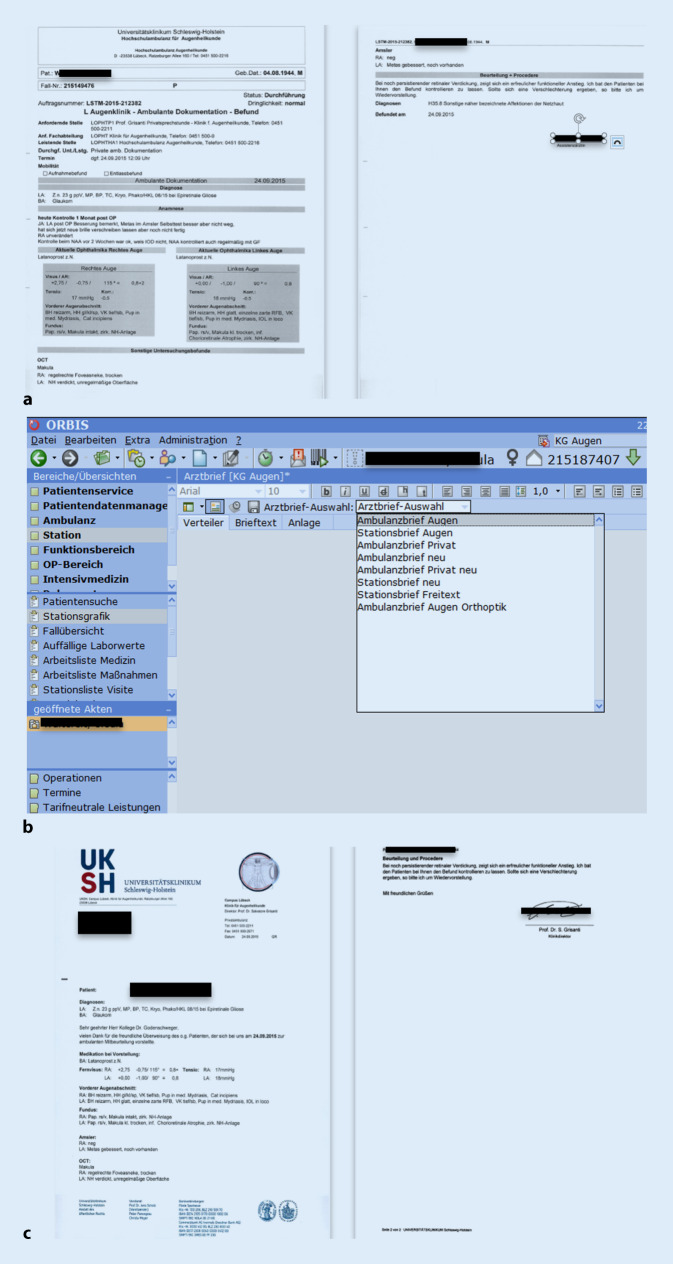

Mit Abschluss der Diagnostik und Therapie, kann aus den erstellten Befundformularen direkt ein Bericht generiert werden (Abb. 6). Dies gilt für stationäre und ambulante Patienten. An die Briefe können alle in der Klinik erhobenen Befunde (z. B. Laborwerte, Radiologiebefunde, Histologiebefunde, Bildgebung etc.) als Anlage beigefügt werden. Die von den Assistenten generierten Briefe werden zur weiteren Signierung z. B. an Oberarzt und/oder Chefarzt gesendet. Jeder Mitarbeiter findet in seiner „Vidierungsliste“ die ihm zugeordneten Briefe und Behandlungsformulare und kann diese so digital weiterbearbeiten und im letzten Schritt digital signieren. Anschließend werden die Briefe zentral im Sekretariat ausgedruckt und versendet. Die Kommunikation mit den Zuweisern erfolgt aber zunehmend per Telematikplattform (z. B. KV Safemail, med.netz.nord). Die Einwilligung des Patienten vorausgesetzt, senden diese Plattformen die Dokumente und Informationen automatisch und über sichere Kommunikationskanäle der Kassenärztlichen Vereinigung an die Zuweiser zur Übernahme in deren Arztinformationssysteme. Die Übertragung wird hierbei über verschiedene Exporte als HL7 MDM dem System bereitgestellt. Die Dokumente werden als e‑Arztbriefe nach dem Standard der KV über das KV-Safenet in einer Safe-Mail oder als CGM-Connect-Nachricht übertragen. Diese kann in den meisten Fällen vom Arztinformationssystem des Empfängers direkt importiert werden. Dieses Kommunikationskonzept wird derzeit von 1284 Ärzten in der Region gerne genutzt. Die Ärzte können den Versand wieder abbestellen, was bis heute für die Arztbriefe von keinem Arzt gewünscht ist. Allerdings wurde der Versanddienst von Laborbefunden in einigen Fällen wieder abbestellt.

Die Anbindung an das med.netz.nord System der UKSH ITSG und das Versenden von Informationen über das gesicherte Netz der KV kann hierbei bundesweit erfolgen.

Im Rahmen einer aktuellen Kooperation zwischen dem UKSH und der Fa. Agfa wird derzeit das Modul Engagesuite weiterentwickelt, um einen bidirektionalen Austausch von Daten, Bildern und Terminen zu ermöglichen.

### Papierloses Arbeiten

Bereits im Jahr 2008 wurde begonnen, die Befundformulare zu erstellen und zu implementierten. Die digitale Dokumentation im ORBIS wurde 2009 begonnen. Mit der Einführung der ophthalmologischen Synopsis, der Geräteanbindung und dem Scannen konnte der Schritt zur volldigitalen Akte vollzogen werden. Im klinischen Alltag werden an der Augenklinik des UKSH, Campus Lübeck keine neuen Akten mehr angelegt, keine Altakten mehr routinemäßig rausgesucht und in keinem Arbeitsbereich mehr Papierakten geführt. Die Patientenverwaltung, Befunderhebung, Dokumentation und Brieferstellung erfolgen komplett im ORBIS.

Anfallendes Papier, wie z. B. Einverständniserklärungen, Fremdbefunde oder Checklisten, werden direkt zur betreffenden EPA eingescannt und können über die Synopsis jederzeit eingesehen werden.

## Diskussion und Ausblick

Wie in der Einleitung erwähnt liegen die Nachteile einer Papierakte auf der Hand. Eine Papierakte ist nicht mehr zeitgemäß und eine elektronische Patientenakte (EPA) erforderlich. Die Digitalisierung wird in sämtliche medizinische Bereiche eindringen und kein Arzt ohne EPA arbeiten können [9].

Die Vorteile einer EPA liegen nicht nur im Bereich des klinischen Alltages. Bereits seit einigen Jahren wird in Großbritannien durch das gezielte Auswerten von Daten aus den EPA die Qualität der Krankenversorgung überprüft, und zudem dienen diese Daten als fundierte Grundlage für weiterführende Forschungsarbeit [3]. Eine Umfrage unter 137 Augenkliniken in Großbritannien aus dem Jahr 2014 stellte jedoch dar, dass zu dem Zeitpunkt nur 45,3 % der Kliniken eine EPA nutzen, auch wenn bei einem Großteil der anderen Kliniken eine Einführung geplant sei. Weiter zeigte sich, dass 87 % dieser Nutzer ein Drittsystem verwenden, nur 2,2 % der befragten Kliniken arbeiten mit einer in das hauseigene IT-System implementierten EPA [7].

In Deutschland erfolgte 2014 eine Umfrage über die Vereinigung Ophthalmologischer Leiter. Nur wenige Universitätsklinika in Deutschland verwendeten zu diesem Zeitpunkt eine EPA. Derzeit gibt es keine Umfrage, die das aktuelle Bild zeigt, es ist aber von einer Verbesserung der Gesamtsituation auszugehen.

Ein Hauptproblem der Konzipierung einer umfänglichen EPA ist das Einbinden der multiplen Untersuchungsergebnisse [2, 7]. Insbesondere in der Ophthalmologie besteht ein hoher Bedarf an Visualisierung und Einbindung von selbst generierten Bilddaten [6]. Aktuell gibt es einige Anbieter, die sich auf die ophthalmologische Patientendokumentation (z. B. IFA Systems AG, Frechen; Arztservice Wente GmbH, Darmstadt; Medisoft Ltd., Leeds, UK) spezialisiert haben und fertige Subsysteme anbieten. Diese Subsysteme können mit den an den Kliniken genutzten KIS über die HL7-Schnittstelle kommunizieren. Es werden fertige Softwarelösungen angeboten, die zumeist noch weiter individualisiert und an die an den Kliniken genutzten medizinischen Geräte angepasst werden können. Entwickelt wurden diese Systeme primär für den niedergelassenen Bereich. Mittlerweile wurden diese auch in großen Kliniken eingeführt [1, 11]. Dennoch lassen sich die Nachteile dieser Subsysteme oder „Insellösungen“ nicht von der Hand weisen. Zum einen sind hohe Anschaffungs- und Erhaltungskosten zu nennen, zum anderen die an den meisten Kliniken strikte Reglementierung von Drittsoftware. Weiterhin sind Nachteile im Datenschutz, der Nachvollziehbarkeit der Dateneingabe und der Datensicherung in Kauf zu nehmen. Mit dem Subsystem kann kein uneingeschränkter bidirektionaler Informationsfluss zwischen dem KIS und der EPA hergestellt werden. Entsprechend muss für bestimmte Aspekte weiterhin das Hauptsystem genutzt werden. Häufig wird in diesem Setting z. B. der OP-Plan im Hauptsystem geführt, damit die anästhesiologische Abteilung Zugriff darauf hat. In diesen Fällen wird bei einer ganz elementaren Funktion eine doppelte Aktenführung durchgeführt (im Hauptsystem und im Subsystem). Das ist nicht nur unpraktisch, sondern stellt auch eine potenzielle Fehlerquelle dar.

Durch eine in das KIS implementierte EPA hat man die gesamte Patientendokumentation in einem System. Man benötigt keine Schnittstelle zu einem Drittanbieter und kann somit einen potenziellen Informationsverlust reduzieren. Das Back-up-System erfüllt höchste Sicherheitsstandards. Der Datenschutz ist erfüllt. Die Nachvollziehbarkeit der Dateneingabe und Dateneinsicht ist gewährleistet. Man kann von jedem Arbeitsplatz der Abteilung und des Gesamtklinikums die volle Funktionalität der EPA nutzen. Insbesondere administrative und interdisziplinäre Abläufe, wie z. B. die OP-Planung, die ambulante Terminplanung (auch gemeinsam mit anderen Fachbereichen), die Anästhesieplanung und -freigabe, sowie die digitale Fieberkurve auf den Stationen, sind einfacher darzustellen. Die gemeinsame (Ärzteschaft und Pflege) Nutzung des gleichen Systems vereinfacht das Arbeiten, vermeidet Schnittstellen und reduziert potenzielle Fehlerquellen.

Dass die EPA bei uns in der jetzigen Form genutzt werden konnte, basiert auf einer kontinuierlichen Entwicklungsarbeit, die hauptsächlich aus Softwareentwicklung und -anpassung bestand. Durch die lange Anbindungsphase (seit 2009) konnte auf die nachträgliche Digitalisierung der Patientenakte (z. B. durch Aktenscan) verzichtet werden. Ein weiterer Vorteil des langsamen Überganges war, dass die Mitarbeiter sukzessive an das System herangeführt wurden und die Umstellung dadurch gut gelang.

Das von uns entwickelte System kann mit wenigen Anpassungen im jedem Agfa-ORBIS-System installiert und genutzt werden. Da Agfa-ORBIS aktuell der Marktführer im Bereich der KIS ist [8], kann diese Lösung in vielen Kliniken eingesetzt werden.

## Fazit für die Praxis

Zusammenfassend lässt sich an diesem Beispiel veranschaulichen, dass durch eine enge Zusammenarbeit des medizinischen und informationstechnologischen Personals ein deutlicher Mehrwert geschaffen wurde. Es konnte auf ein kostspieliges Drittsystem verzichtet werden. Zudem ist die EPA im KIS das einzige System mit einer lückenlosen, fächerübergreifenden ubiquitären Dokumentation.
